# The first report of the seroprevalence of antibodies against *Bartonella* spp. in water buffaloes (*Bubalus bubalis*) from South Thailand

**DOI:** 10.14202/vetworld.2021.3144-3148

**Published:** 2021-12-24

**Authors:** Sumalee Boonmar, Phirabhat Saengsawang, Watcharapong Mitsuwan, Decha Panjai, Kamchai Kidsin, Chalutwan Sansamur, Ittidet Wichianrat

**Affiliations:** 1 Akkhraratchakumari Veterinary College, Walailak University, Thasala, Nakhon Si Thammarat, 80160, Thailand; 2 Research Center of Excellence in Innovation of Essential Oil, Walailak University, Nakhon Si Thammarat 80160, Thailand; 3 Department of Medical Sciences, National Institute of Health, Ministry of Public Health, Nonthaburi, 11000, Thailand; 4 Animal Health Section, The Eight Regional Livestock Development, Surat Thani 84000, Thailand

**Keywords:** *Bartonella henselae*, *Bartonella tamiae*, *Bartonella vinsonii* subspp. *berkhoffii*, immunofluorescence assay, seroprevalence, water buffaloes

## Abstract

**Background and Aim:**

Bartonellosis is an emerging worldwide zoonosis caused by bacteria belonging to the genus *Bartonella*. Several studies have been conducted on the prevalence of *Bartonella* infections from animals and humans, including reports from wild and domestic ruminants. However, there has been only one report of *Bartonella* infection in water buffaloes from the northeastern part of Thailand. Moreover, the seroprevalence of *Bartonella* spp. in water buffaloes still remains unknown. This study was conducted to explore the prevalence of *Bartonella* spp. among water buffaloes from South Thailand using molecular and serological techniques.

**Materials and Methods:**

A total of 312 samples (156 blood and 156 sera) of 156 water buffaloes from 29 farms in Phatthalung Province, South Thailand, were collected from January to March 2021. All samples were screened for *Bartonella* spp. using polymerase chain reaction and indirect immunofluorescence assay.

**Results:**

The seroprevalence of antibodies against three *Bartonella* spp. was 16.03% (25/156, 95% confidence interval: 10.65-22.74%), and among 25 water buffaloes with seroprevalence, 56%, 20%, and 24% were positive for antibodies against *Bartonella* *henselae*, *Bartonella* *vinsonii* subspp. *berkhoffii*, and *Bartonella* *tamiae*, respectively. No significant difference was detected among seroprevalence, gender, age, and ectoparasite infestation.

**Conclusion:**

This is the first report of the seroprevalence of antibodies against *B. henselae*, *B. vinsonii* subspp. *berkhoffii*, and *B. tamiae* in water buffaloes from South Thailand. Further studies are required on the epidemiology of *Bartonella* infection among water buffaloes, related personnel, and ectoparasites.

## Introduction

Bartonellosis, an emerging zoonosis, is responsible for causing a variety of clinical syndromes in humans and animals and is associated with significant morbidity and mortality in humans worldwide [1,2]. A microorganism in the genus *Bartonella* is the major causative agent of this disease. *Bartonella* spp. are Gram-negative fastidious oval bacteria that infect mammalian erythrocytes and endothelial cells of the host. It is well known that some *Bartonella* spp. are the cause of a wide spectrum of human illnesses such as cat scratch disease, chronic bacteremia, fever, and endocarditis [2]. It has been reported that human Bartonellosis is caused by *Bartonella* *henselae*, *Bartonella* *bacilliformis*, and *Bartonella* *quintana* [3]. Furthermore, various species of the pathogens, including *Bartonella bovis*, *B. henselae*, and *Bartonella* *clarridgeiae*, have been detected from several animals, including buffalo, dog, and cat [4,5]. Mammalian animals such as buffaloes are also considered reservoir hosts and can transmit pathogens to humans.

Water buffaloes (*Bubalus bubalis*) are one type of cattle belonging to the family Bovidae and subfamily Bovinae [6]. Buffaloes are agriculturally and economically important animals in several countries, including Thailand. They are commonly infested by numerous ectoparasites that are considered vectors of hemoparasites, including *Bartonella* spp. The major transmission route of this bacterium is through biting from the arthropod vectors such as ticks, mites, lice, fleas, and flies to animals and humans [7,8]. Previous studies have reported the infection of *Bartonella* spp. in buffaloes and the detection of bacterial species from buffalo samples [4,9]. There is also a report of *Bartonella* infection in water buffaloes from the northeastern and middle parts of Thailand [4]. However, the seroprevalence of *Bartonella* spp. in water buffaloes in Thailand still remains unknown.

Therefore, the aim of the study was to explore the seroprevalence of *Bartonella* spp. in water buffaloes in 29 farms in South Thailand using polymerase chain reaction (PCR) and indirect immunofluorescence assay (IFA).

## Materials and Methods

### Ethical approval

The study was approved by the Institutional Animal Care and Use Committee of the National Institute of Health (NIH), Thailand.

### Study period and location

The study was conducted from January to March 2021. The samples were collected from Phatthalung providence of Thailand. The samples were processed at Department of Medical Sciences, NIH Laboratory, Ministry of Public Health, Nonthaburi, Thailand.

### Sample collection

We calculated the sample size based on 4124 water buffaloes using the Epitools program (www.epitool.net) with 95% confidence interval (CI), 0.05 precision, and 0.1 prevalence [4] in Thailand. The result showed that 133 samples needed to be collected. Overall, 312 blood and sera samples of 156 water buffaloes from 29 farms in Phatthalung Province were collected. Approximately 10 μl of blood was aseptically collected from the jugular vein of each water buffalo and transferred to EDTA-containing collection tubes. In addition, 5 ml of blood was collected into a plain tube, and the obtained serum was stored in a sterile microcentrifuge tube. The gender, age, and health status, including ectoparasite infestation of each water buffalo were recorded. Samples were transported to the Department of Medical Sciences, NIH Laboratory, under chilled conditions and stored at −20°C until processing.

A total of 156 water buffaloes were investigated in this study, comprising 106 females (67.9%) and 50 males (32.1%). These buffaloes were divided into two age groups as follows: 63 water buffaloes (40.4%) were aged ≤2 years and the remaining 93 buffaloes (59.6%) were aged >2 years. Furthermore, 109 (69.87%) buffaloes were infested with ectoparasites (lice).

### Isolation of *Bartonella*

Isolation of *Bartonella* was done according to a previously described method [10] with modification. Briefly, frozen blood samples were thawed at 25ºC, and then, 200 μL of blood was centrifuged. The sediment was mixed with the same volume of medium 199 supplemented with sodium pyruvate and fetal bovine serum (Life Technologies, USA). The mixture was then plated onto brain heart infusion agar (BHIA, Difco, USA) plates containing 5% defibrinated rabbit blood. The plates were incubated at 35°C under 5% CO_2_ for 2-4 weeks. This showed the presence of Gram-negative coccobacilli growing in small, rough, and grayish colonies that required long culture periods, which were tentatively considered as *Bartonella* organisms. The bacteria were subcultured on the culture media for further characterization.

### DNA extraction and PCR amplification

*Bartonella* DNA from the samples was identified by the detection of genomic DNA using specific primers by PCR as described previously by Pangjai *et al*. [10]. The genomic DNA was extracted from each isolate using InstaGene Matrix (Bio-Rad, Hercules, USA). Primers targeting the β-subunit of RNA polymerase (rpoB) [11] (primer pair sequences 5́ CGCATTGGCTTACTTCGTATG 3́ and 5́ GTAGACTBATTAGAACGCTG 3́) and citrate synthase (*gltA*) [12] (primer pair sequences 5́ AATGCAAAAAGAACAGTAAACA 3́ and 5́ GGGGACCAGCTCATGGTGG 3́) were used for PCR analysis. The thermal cycling conditions of PCR included a first denaturation step at 94°C for 2 min, followed by 35 cycles of 94°C for 30 s, 53°C for 30 s, and 72°C for 1 min, with a final step of 72°C for 7 min. Positive and negative controls were included in each experiment. Finally, 10 μL volume of each PCR product was subjected to electrophoresis on 1.5% agarose gels containing ethidium bromide and visualized on a UV transilluminator. The length of PCR products, 825 bp (rpoB primers) and 379 bp (gltA primers) should be observed.

### Indirect IFA

Buffalo sera were screened for antibodies against *Bartonella* spp. using IFA as described previously by Pangjai *et al*.[10]. Briefly, 10 μl of blood was centrifuged at 1500× g for 15 min to collect serum. Then, the antigens of *Bartonella* spp., including *B. henselae*, *B. vinsonii* subspp. *berkhoffii*, and *Bartonella* tamiae (provided by the US-CDC), were fixed on the slides. Next, 10 μL of diluted serum (diluted in PBS containing 5% skim milk) was placed into the test holes, and the slides were incubated at 37°C for 1 h in a humid chamber. Then, the slides were washed twice with PBS for 15 min. Fluorescein-conjugated goat anti-bovine IgG (KPL Antibody and Conjugates Products, SeraCare Corp, USA) was diluted 1:800 in PBS with 0.001% Evans blue, and 10 μL of the mixture was applied into each hole. The slides were incubated for 1 h at 37°C, washed twice with PBS for 15 min, washed again with double-distilled water for 10 min, and then dried before examination under a fluorescence microscope. The intensity of the bacillus-specific fluorescence was scored subjectively from +1 to +4, and the fluorescence score of +2 at a dilution of 1:16 was considered to be positive. Serum samples were screened at 1:16 dilution, and the positive sample (at 1:16 dilution) was titrated in a series of 2-fold dilutions up to 1:1024. Positive and negative controls were included in the study.

### Statistical analysis

The odds ratio was calculated using “epiR” package embedded in R program, version 4.02[13]. The observed differences were considered to be statistically significant at p≤0.05.

## Results

Overall, 16.03% (25/156, 95% CI: 10.65-22.74%) of the water buffalo sera samples were positive for three *Bartonella* antigens at the cutoff titer of 1:16. Table-1 shows the seroprevalence of antibodies against three *Bartonella* antigens, wherein among the 25 positive buffaloes, 56% (14/25) were positive for *B. henselae* antigen only, 20% (5/25) were positive for *B. vinsonii* subspp. *berkhoffii* antigen only, and 24% (6/25) were positive for *B. tamiae* antigen only. Interestingly, 1 (4%) of the 25 buffaloes was found to be positive for *B. vinsonii* subspp. *berkhoffii* at the cutoff titer of 1:64. The comparisons of *Bartonella* spp. seroprevalence assessed by IFA among gender, age, and ectoparasite infestation using univariate analysis are summarized in Table-2. No significant differences were observed among the seroprevalence, gender, age, and ectoparasite infestation. As shown in Table-3, the seroprevalence of antibodies at the herd level was 34.5% (10/29). Among the 29 farms, 25 positive buffaloes were detected in 10 farms, and each farm contained buffaloes that had positive antibodies against *Bartonella* spp. Especially in farm 2, there was one buffalo harboring two antibodies against *B. henselae* and *B. vinsonii* subspp. *berkhoffii*. However, PCR analysis of any sample did not reveal *Bartonella* DNA.

**Table 1:** Seroprevalence of antibodies against *B. henselae, B. vinsonii subspp. berkhoffii,* and *B. tamiae* by IFA.

Antibody titer	*B. henselae*	*B. vinsonii* subspp. *berkhoffii*	*B. tamiae*
1:16	14/25 (56%)	3/25 (12%)	6/25 (24%)
1:32	-	1/25 (4%)	-
1:64	-	1/25 (4%)	-

*B. henselae=Bartonella henselae, B. vinsonii=Bartonella vinsonii, B. tamiae=Bartonella tamiae,* IFA=Immunofluorescence assay

**Table 2:** Statistical association between the seroprevalence of antibodies against *B. henselae, B. vinsonii subspp. berkhoffii, B. tamiae,* and different factors (univariate analysis).

Factor	Total	*Bartonella* spp. (25/156)			*B. henselae* (14/156)			*B. vinsonii* subspp*. berkhoffii* (5/156)			*B. tamiae* (6/15 6)		
			
		P	N	p	P	N	p	P	N	p	P	N	p
Gender													
Female	106	17	89	1	10	96	1.00	4	102	1	3	103	0.39
Male	50	8	42		4	46		1	49		3	47	
Age													
≤2 y	63	14	49	0.13	8	55	0.29	3	60	0.39	3	60	0.69
>2 y	93	11	82		6	87		2	91		3	90	
Ectoparasite													
Yes	109	19	90	0.62	10	99	1.00	4	105	1	5	104	0.67
No	47	6	41		4	43		1	46		1	46	

P=Positive, N=Negative, *P=* p-value. *B. henselae=Bartonella henselae, B. vinsonii=Bartonella vinsonii,*

*B. tamiae=Bartonella tamiae*

**Table 3:** Herd seroprevalence and titer of antibodies against *B. henselae, B. vinsonii* subspp. *berkhoffii*, and *B. tamiae* in positive buffaloes.

Farm	Number of buffaloes	Number of positive buffaloes (%)	Titer of *B. henselae*	Titer of *B. vinsonii* subspp. *berkhoffii*	Titer of *B. tamiae*
1*	11	4 (36.4)	1:16	-	1:16, 1:16, 1:16
2*	6	2** (33.3)	1:16, 1:16	1:16	-
3*	6	5 (83.3)	1:16, 1:16, 1:16	1:32	1:16
4*	14	6 (42.9)	1:16, 1:16, 1:16, 1:16	-	1:16, 1:16
5	11	-	-	-	
6	12	-	-	-	
7	12	-	-	-	
8*	12	2 (16.7)	-	1:64, 1:16	-
9	9	-	-	-	-
10*	7	1 (14.3)	1:16	-	-
11	4	-	-	-	-
12	8	-	-	-	-
13	2	-	-	-	-
14	1	-	-	-	-
15	2	-	-	-	-
16	2	-	-	-	-
17	2	-	-	-	-
18	1	-	-	-	-
19	2	-	-	-	-
20*	2	1 (50)	1:16	-	-
21	1	-	-	-	-
22*	1	1 (100)	1:16	-	-
23	1	-	-	-	-
24	9	-	-	-	-
25*	3	1 (33)	1:16	-	-
26	3	-	-	-	-
27	6	-	-	-	-
28	4	-	-	-	-
29*	2	1 (50)	-	1:16	-

* Positive farms containing positive water buffaloes, **one buffalo containing two antibodies against to *B. henselae* and *B. vinsonii* subspp. *berkhoffii. B. henselae=Bartonella henselae, B. vinsonii=Bartonella vinsonii, B. tamiae: Bartonella tamiae*

## Discussion

This is the first report of the detection of *Bartonella* spp. in water buffaloes using IFA. Our result showed that 16% (25/156, 95% CI: 10.65-22.74%) of the water buffaloes harbored antibodies against three *Bartonella* spp. We suspected that those buffaloes had been infected with *Bartonella* in the past. Some authors had reported that higher antibody titers were associated with bacteremia in animals and lower antibody titers might indicate slight bacteremia [14]. However, in our study, *Bartonella* DNA was not detected using PCR. Due to the limitation of information on the seroprevalence of *Bartonella* antibodies from buffaloes in Thailand, we could not compare our results with other studies.

Recently, studies have reported the high prevalence of *B. bovis*, *Bartonella* *chomellii*, *Bartonella* *schoenbuchensis*, *Bartonella* *capreoli*, and *Bartonella* *melophagi* infections in ruminants [15-18]. *B. bovis* infection was reported in 6.8% of water buffaloes in Thailand [4] and in 4.1% of wild African buffaloes (*Syncerus caffer*) in Mozambique, Africa [9], using molecular technique. However, our results showed the presence of antibodies against *B. henselae*, *B. vinsonii* subspp. *berkhoffii*, and *B. tamiae*, and not against *B. bovis*, in the water buffaloes.

A high prevalence of *B. henselae* infection has been reported in dogs and cats as well. Not only *B. henselae* but also *B. vinsonii* subspp. *berkhoffii* was detected in Thai cats [19-21]. Our literature search also showed a report of *B. tamiae* isolated from patients with febrile illness in Thailand who had possible rodent contact and were either dog or cat owners [22]; we also found a report of *Bartonella* spp. isolated from ectoparasites (chigger mites) and ticks of rodents in Thailand [23].

There are also some reports showing that infected arthropods could transmit *Bartonella* spp. to humans, such as *B. henselae* from cat fleas [7,24], *B. quintana* from human body lice [25], and *Bartonella* spp. from cattle lice [26]. Unfortunately, we collected arthropod vectors such as lice from the buffaloes, and we did not identify *Bartonella* pathogens in those lice. Therefore, the mechanism through which the buffaloes acquired antibodies against *Bartonella* spp. remains unknown. It is necessary to clarify the knowledge gaps concerning the distribution of this bacterium, the genetic diversity, and the transmission mode among water buffaloes, related personnel, and ectoparasites.

## Conclusion

This is the first seroprevalence report of antibodies against *B. henselae*, *B. vinsonii* subspp. *berkhoffii*, and *B. tamiae* in water buffaloes from South Thailand. Further epidemiological studies should be conducted, including extensive surveys and additional studies of ectoparasites covering more areas and large samples sizes, to understand the risk factors for this disease among water buffaloes, related personnel, and ectoparasites.

## Authors’ Contributions

KK, CS, WM, and IW: Collected the samples. DP: Provided technical help during the experiments. PS: Did the statistical analysis. SB: Designed the experiments and drafted and revised the manuscript. All authors read and approved the final manuscript.
